# Radio-Resistance and DNA Repair in Pediatric Diffuse Midline Gliomas

**DOI:** 10.3390/cancers12102813

**Published:** 2020-09-30

**Authors:** Henriette Pedersen, Kjeld Schmiegelow, Petra Hamerlik

**Affiliations:** 1 Brain Tumor Biology, Danish Cancer Society Research Center, Strandboulevarden 49, DK-2100 Copenhagen, Denmark; henped@cancer.dk; 2 Department of Paediatrics and Adolescent Medicine, Rigshospitalet, Copenhagen University Hospital, Juliane Maries Vej 8, DK-2100 Copenhagen, Denmark; kjeld.schmiegelow@regionh.dk; 3 Department of Clinical Medicine, Faculty of Health and Medical Sciences, University of Copenhagen, Blegdamsvej 3B, DK-2200 Copenhagen, Denmark; 4 Department of Drug Design and Pharmacology, Faculty of Health and Medical Sciences, University of Copenhagen, Jagtvej 160, DK-2100 Copenhagen, Denmark

**Keywords:** radio-resistance, pediatric high-grade gliomas, H3K27M mutation, DNA damage response

## Abstract

**Simple Summary:**

Approximately 50% of high-grade gliomas (HGG) in children are diffuse midline gliomas (DMGs), which carry the worst prognosis of all HGG, with a 2-year survival of less than 10%. DMGs are characterized by H3K27M mutation, rampant genomic instability, infiltrative growth, and radio-resistance. Recent large-scale profiling studies have identified some of the key molecular drivers underpinning DMG biology and therapeutic resistance. Here, we provide a comprehensive overview of studies that focus on DMG in the context of radio-resistance. We speculate that the aberrant activation of DNA damage response pathway (DDR) represents a druggable vulnerability, which could be leveraged to radio-sensitize DMGs.

**Abstract:**

Malignant gliomas (MG) are among the most prevalent and lethal primary intrinsic brain tumors. Although radiotherapy (RT) is the most effective nonsurgical therapy, recurrence is universal. Dysregulated DNA damage response pathway (DDR) signaling, rampant genomic instability, and radio-resistance are among the hallmarks of MGs, with current therapies only offering palliation. A subgroup of pediatric high-grade gliomas (pHGG) is characterized by H3K27M mutation, which drives global loss of di- and trimethylation of histone H3K27. Here, we review the most recent literature and discuss the key studies dissecting the molecular biology of H3K27M-mutated gliomas in children. We speculate that the aberrant activation and/or deactivation of some of the key components of DDR may be synthetically lethal to H3K27M mutation and thus can open novel avenues for effective therapeutic interventions for patients suffering from this deadly disease.

## 1. Diagnosis and Treatment

Despite the fact that primary pediatric central nervous system (CNS) tumors are rare (approximately 3.2 cases per 100,000 children per year [1]), they represent the most common solid tumor in childhood and account for the highest number of cancer-related deaths in children [2,3]. Among these malignancies, diffuse intrinsic pontine gliomas (DIPG) are the most aggressive subset with an incidence of less than one case per 100,000 children per year [1]. The anatomic location and infiltrative nature of DIPGs precludes successful resection and leads to a variety of progressive neurological deficits during the short life span, ultimately resulting in death [4]. The median age at diagnosis is seven. The median progression-free survival is seven months and once progression is diagnosed, patients die within 2–4 months [5,6].

**Figure 1:**
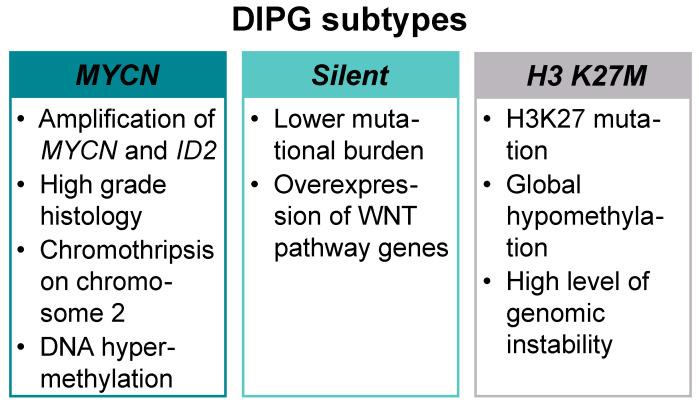
Molecular subtypes of diffuse intrinsic pontine glioma (DIPG). DIPGs can be divided into three distinct molecular subtypes: *MYCN*, *Silent*, and *H3 K27M*.

Recent technological advancement in diagnostics, especially the use of whole genome sequencing (WGS) at diagnosis or post-mortem, has mapped the complexity of pediatric high-grade gliomas (pHGG) at the genomic and epigenomic level, showing that recurring somatic mutations in histone H3 and other key signaling pathways underpin the growth, proliferation, survival, and therapeutic resistance of these tumors [6,7]. Three molecular subtypes—*MYCN*, *Silent*, and *H3 K27M*—have been defined on the basis of the integrative analysis of DNA methylation profiling, WGS, and histopathological and clinical data (Figure 1).

A high-grade histology, chromothripsis (high frequency of chromosomal rearrangements acquired in one or more regions) on chromosome 2, DNA hypermethylation, and high-level amplification of MYCN proto-oncogene bHLH transcription factor (*MYCN)* and inhibitor of DNA binding 2 (*ID2)* are the hallmarks of *MYCN* subtype. The *H3 K27M* subtype is the most prevalent in DIPG and presents with H3K27 mutation, global hypomethylation, and multiple concurrent mutations (Table 1) [8,9].

The *Silent* subtype carries lower mutational burden than the other two and shows overexpression of wingless-related integration site (WNT) pathway genes. Patients in the *silent* subgroup are significantly younger compared to the other two subgroups (5 years for *silent*, 6.3 years for *MYCN*, and 7 years for *H3 K27M*) and present with a low-grade astrocytoma histology [8,10]. In a more recent study, Mackay et al. defined the following molecular subgroups: H3G34R/V, H3K27M, high grade glioma wildtype (HGG WT), isocitrate dehydrogenase 1 (IDH1), low-grade glioma (LGG)-like, pleomorphic xanthoastrocytoma (PXA)-like, and “other”. As reported previously, these subgroups differed in anatomical location, age at diagnosis, and overall survival, with the LGG-like group representing a younger cohort and excellent prognosis [11].

**Figure 2:**
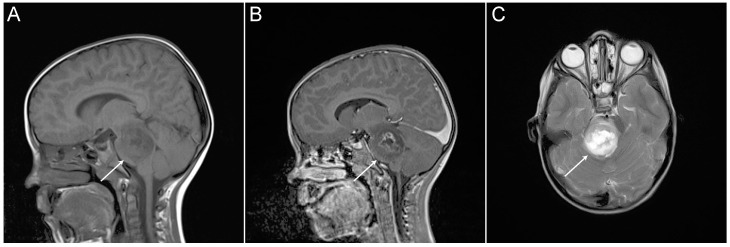
Magnetic resonance imaging (MRI) of DIPG tumor from an individual patient (white arrows). (**A**) Sagittal T1-weighted. (**B**) Sagittal T1-weighted. (**C**) Axial T2-weighted.

In 2016, the World Health Organization (WHO) reclassified CNS tumors to take account of molecular pathology, with this including definition of a new entity—histone 3 K27M-mutated diffuse midline gliomas (DMGs)—which encompass about 80% of DIPGs [12,13]. Clinical presentation, history, physical neurological examination, and magnetic resonance imaging (MRI) are generally used in diagnosis of DMGs. Typical radiographic characteristics of DIPG on MRI include a T1 hypointense, T2 hyperintense tumor centered in and involving > 50% of the pons, with absence of or irregular gadolinium enhancement (Figure 2) [9,14]. Given the infiltrative nature of these tumors, surgical intervention is substantially limited and therapy has been traditionally instituted on the basis of imaging data, usually without histopathological verification. A series of conflicting studies cited morbidity rates between 0% and 10% and mortality rates between 0% to 3% to be associated with stereotactic biopsy procedure. However, when biopsy data on pediatric patients were extracted, the diagnostic yield ranged from 96% to 100%, with no mortality and morbidity less than 5% for the largest series [15]. Most importantly, molecular analysis from biopsy material has greatly increased our chances for a better understanding of the unique biological behavior of DMGs [16,17].

The current standard of care consists of fractionated radiation (delivered most commonly using photon radiation therapy (RT)), typically with a total dose of 54–60 Gy delivered with a daily fraction of 1.8–2 Gy over 6 weeks. Steroids are often used during the course of RT to relieve symptoms of peri-tumoral oedema [16]. Radiation therapy allows transient control of the disease or even symptomatic relief, but recurrence is inevitable. Of note, there is some evidence suggesting a certain degree of radio-sensitivity remains at this stage, since some DMG patients were found to benefit from re-irradiation [18]. Re-irradiation with approximately 25 Gy over 10 fractions has been reported to be safe and suggests a modest survival benefit, as well as stabilization and/or improvement in symptoms [13].

Chemotherapy (such as alkylating agent temozolomide (TMZ), carboplatin, cisplatin, or irinotecan) alone, or as an adjuvant to RT, has not been associated with any additional survival benefit [19,20]. Numerous regimens of either a mono- or combinational chemotherapy have been trialed with overall poor outcome. Agents targeting other molecular pathways, such as erlotinib (tyrosine kinase EGFR inhibitor) and bevacizumab (humanized mouse antibody targeting vascular endothelial growth factor A (*VEGFA*)) have also been proven ineffective, whether administered alone or in combination with RT [8]. Similarly, radio-sensitizers (e.g., carbogen, motefaxin gadolinium) did not improve the survival of DMG patients [21,22]. Currently in clinical trials for DMGs are inhibitors of histone deacetylases (HDAC), which emerged from functional screen data as well as the observation of consistently elevated histone acetylation in a number of studies [23].

The blood–brain barrier (BBB) is thought to be one of the key reasons why DMG patients respond poorly to chemotherapy and other targeted therapies. In DMGs, the BBB is frequently intact, as evidenced by the lack of gadolinium enhancement. Interestingly, some studies suggest that the brainstem region may have even more robust BBB compared to other areas of the brain [24]. Numerous methods have been developed in an attempt to bypass this barrier—among these, convention-enhanced delivery (CED) or the use of nanoparticles are being extensively tested in pre-clinical as well as the clinical setting [25,26]. The CED approach utilizes a catheter to deliver drugs directly to the target region [26]. There are active clinical trials for DMGs that are exploring the delivery of the conventional chemotherapeutic irinotecan (NCT03086616) and a radioactive iodine-labeled monoclonal antibody omburtamab (NCT01502917) via CED [27,28]. The National Clinical Trial NCT03566199 explores the CED approach in combination with HDAC inhibitor panobinostat in a nanoparticle formulation (MTX110) [29].

Despite intense scientific effort and recent developments in the clinical trial setting, the fact that treatment options for DMG patients are limited stresses the urgent need to develop novel therapeutics and effective delivery mechanisms for this patient group.

## 2. Mutations and Key Signaling Genes in DMG

Somatic mutations in histone genes in pHGG were first reported by two groups [6,7]. Wu et al. performed WGS on seven DIPGs and targeted sequencing on an additional cohort of 43 DIPGs and 36 non-brainstem pediatric glioblastomas (pGBMs). They found that 78% of DIPGs and 22% of non-brainstem pGBMs contained heterozygous mutations in H3 histone family member 3A (*H3F3A*) and histone cluster 1 H3B (*HIST1H3B*), respectively [6]. The study by Schwartzentruber et al. included 48 pGBMs and found that somatic mutations of histone genes, including H3.3K27M and H3.3G34R/V, occurred in 31% of patients [7]. These mutations result in a substitution of histone 3 lysine to methionine at position 27 (H3K27M) or glycine to arginine/valine at position 34 (H3G34R/V). While H3.3K27M-mutated tumors are localized in midline structures (e.g., brainstem, thalamus, cerebellum, and spine), the H3.3G34R/V-mutated tumors locate exclusively in cerebral hemispheres. The histone variant H3.1 (*HIST1H3B* gene) was found mutated and solely associated with DMG brainstem tumors (H3K27M), occurring in 18% of cases [6,30]. The H3K27M mutation in H3.3 histone occurs with alterations in the p53 pathway, mostly tumor suppressor protein p53 (*TP53*)- and protein phosphatase magnesium-dependent 1 delta (*PPM1D*)-inactivating mutations, while H3.1 histone mutation associates with activating mutations in the activin A receptor type 1 (*ACVR1A)* gene as well as whole chromosome 2 gain and chromosome 16q loss [7,31,32]. DNA topoisomerase III alpha (*TOP3A)* amplification is *TOP3A* alterations that were mutually exclusive with *ATRX* deletion/mutations found in H3.3K27M DIPGs [11,33].

**Figure 3:**
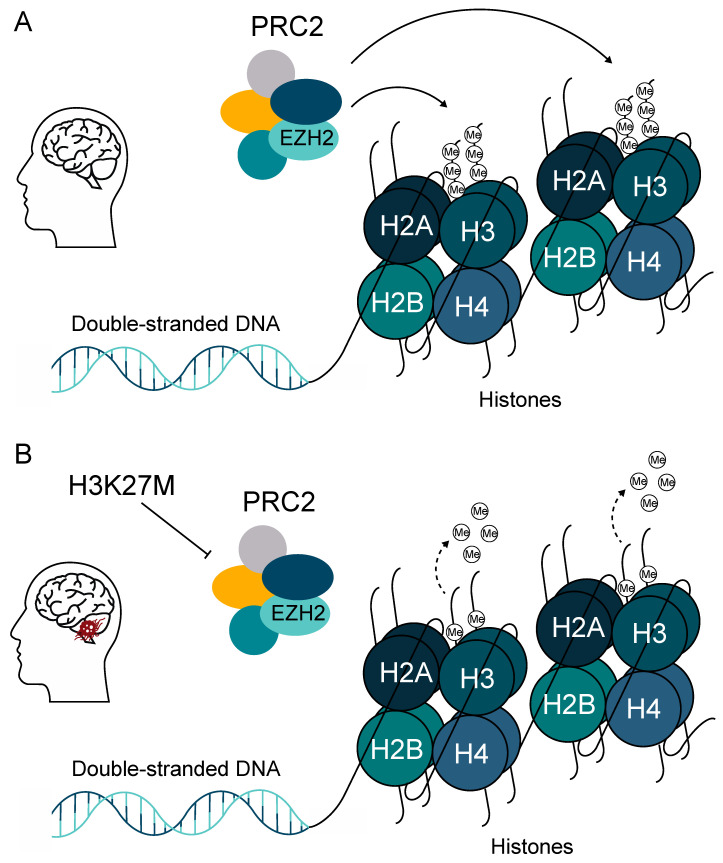
H3K27M is the major oncogenic driver in pediatric high-grade gliomas (pHGG). (**A**) The polycomb repressive complex 2 (*PRC2)* complex mediates the di- and trimethylation of H3K27 in healthy brains. (**B**) The H3K27M mutation in diffuse midline gliomas (DMG) leads to global loss of di- and trimethylation of histone H3K27 (H3K27me2 and H3K27me3) by inactivating the *PRC2* complex.

A study by Buczkowicz et al. (2014) revealed that DIPG outcome correlated more accurately with H3 mutation status than with WHO grade, with the overall survival rate for WHO grade II H3.3-mutated patients being comparable with that of WHO grade IV GBM tumors [10]. The oncogenesis of DMGs is driven by the lysine-to-methionine substitution at position 27 of the regulatory tail of histone variants *H3F3A*, *HIST1H3B/C*, and *HIST2H3A*. This mutation is dominant negative and heterozygous, whereas each histone H3 variant mutated at K27 drives a specific oncogenic program [34]. H3K27M mutation drives the global loss of di- and trimethylation of histone H3K27 (H3K27me2/H3K27me3) and affects the enzymatic activity of enhancer of zeste homologue 2 (*EZH2*) [35,36]. *EZH2* is the main component of the polycomb repressive complex 2 (*PRC2*), which under normal conditions mediates the di- and trimethylation on H3K27 (Figure 3) [36]. A recent study by Harutyunyan et al. showed that the removal of H3K27M mutation restores the levels of H3K27me2/H3K27me3 and impairs the tumorigenic capacity of DMG cells in vivo in a mouse model [37]. Interestingly, the authors found that the loss of H3K27me2 and H3K27me3 induces limited transcriptomic changes, while genes that are actually dysregulated are primarily involved in stemness and neurogenesis [37].

Similar to other cancer types, DMGs are characterized by frequent aberrations involving a number of signaling pathways downstream of cell surface receptors. Focal amplifications and mutations to the *PDGFRA* as well as concurrent focal amplifications in other receptor tyrosine kinases (RTKs) such as KIT proto-oncogene receptor tyrosine kinase (*KIT*)/vascular endothelial growth factor receptor 2 (*VEGFR2*)*/MET/*epidermal growth factor receptor (*EGFR2*) are some of the most common genomic events found in DMGs [4]. Although no single event is prevalent in these tumors, studies have identified mutations in both phosphatidylinositol-4,5-bisphosphate 3-kinase catalytic subunit alpha (*PIK3CA*) and phosphoinositide-3-kinase regulatory subunit 1 (*PIK3R1)*, HRas proto-oncogene GTPase (*HRAS*) amplification, and phosphatase and tensin homolog (*PTEN*) loss, jointly contributing to an aberrant activation of the phosphatidyl-inosital-3 kinase (*PI3K*) and/or mitogen-activated protein kinase (*MAPK*) signaling in these tumors [38].

The abrogation of cell cycle checkpoints leading to dysregulated cell cycle kinetics is among the hallmarks of cancer [39]. The H3K27M mutation reportedly results in epigenetic silencing of cyclin-dependent kinase inhibitor 2A (*CDKN2A*), a key regulator of G1/S checkpoint. In addition, essential players in the G1/S checkpoint activation, *CDK4/6*, and associated cyclins have been reported to be amplified in DMGs [40]. Besides the aberrant expression of G1/S checkpoint regulators, DMGs were found to overexpress several key players in the G2/M checkpoint progression such as Wee1 G2 checkpoint kinase (*Wee1*), maternal embryonic leucine zipper kinase (*MELK*), polo-like kinase 1 (*PLK1*), and aurora kinase B (*AURKB*) [41,42,43].

## 3. DNA Repair Response Pathway

Error-free and timely regulation of cell cycle progression is crucial for genome integrity maintenance. Each of the ≈10^13^ cells in the human body receives tens of thousands of DNA lesions per day [44]. These DNA lesions can result from either endogenous (e.g., replication errors, reactive oxygen species (ROS)) or exogenous sources (e.g., ultraviolet light (UV), ionizing radiation (IR), chemical agents) [45]. Exposure to IR inflicts single-stranded DNA breaks (SSBs), double-stranded DNA breaks (DSBs), base damage, and DNA-protein cross-links in the genomic DNA. Among them, DSBs are the most deleterious lesions that, if not repaired correctly, can lead to genomic instability and cell death [46].

**Figure 4:**
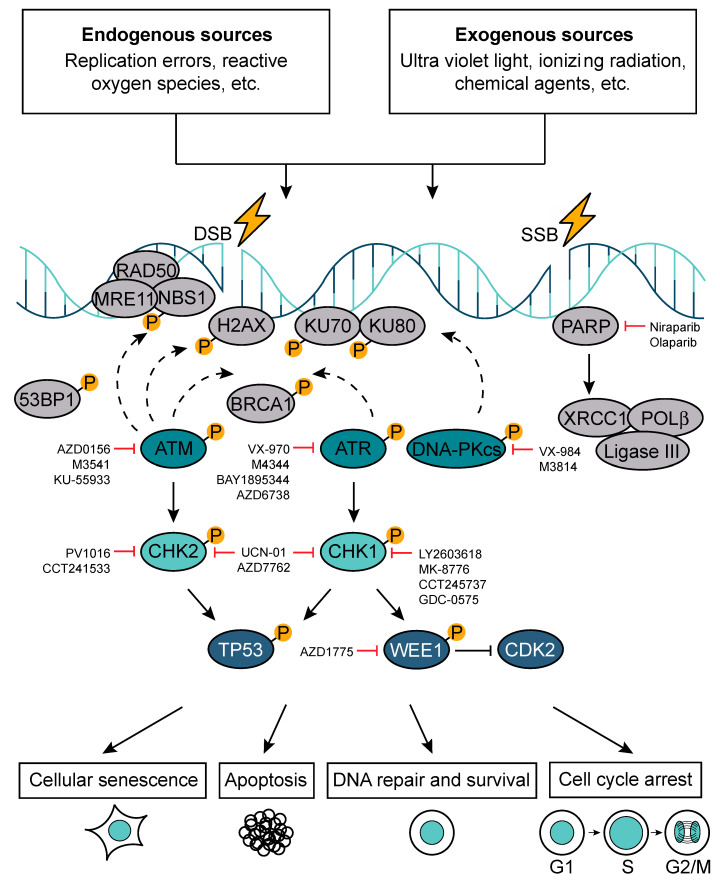
DNA damage response pathway (DDR). In response to DNA damage, which can be induced by the exposure to either endogenous or exogenous factors, cells activate DDR. Depending on the extent and type of DNA damage (single-strand DNA break (SSB) versus double-strand DNA break (DSB)), the DDR can trigger cell cycle arrest, apoptosis, cellular senescence, or DNA repair. A number of DDR components have been successfully targeted in pre-clinical settings.

A sophisticated network of DNA damage response (DDR) systems has evolved to deal with the fundamental problem of maintaining genomic stability (Figure 4). These include a set of DNA repair mechanisms, damage tolerance processes, and cell-cycle checkpoint pathways. Depending on the type of DNA damage and the outcome of DNA repair, DDR activation can lead to cell survival, apoptosis, cell cycle arrest, or cellular senescence (e.g., permanent cell cycle arrest) [44,47].

The protein kinases ataxia telangiectasia mutated (*ATM*), ataxia telangiectasia and rad3 related (*ATR*), and DNA-dependent protein kinase catalytic subunit (*DNA-PKcs*) are the key regulators of DDR. *ATM* and *ATR* activation triggers an activatory phosphorylation cascade, which results in the phosphorylation of DDR mediators (e.g., p53-binding protein 1 (*53BP1*), H2A histone family member X at Serine 139 (*H2AXSer139 or γH2AX*), breast cancer type 1 susceptibility protein (*BRCA1*)) and downstream kinases checkpoint kinases 1/2 (*Chk1*/*Chk2*). *Chk1* and *Chk2* then in turn phosphorylate downstream effectors such as *TP53*. *DNA-PKcs* activation leads to the formation of a complex with the Ku heterodimer (Ku70/Ku80), playing an important role in non-homologous end-joining (NHEJ) [48]. There are two major pathways devoted to DSBs repair: NHEJ and homologous recombination DNA repair (HR), which operate in a cell-cycle phase-dependent manner. NHEJ can occur in all phases of the cell cycle (dominates in G1) and is error-prone because it directly ligates broken DNA ends. The error-free repair by HR is restricted to S and G2 phase and it uses sister chromatids as a template for the repair [49]. The G1/S checkpoint is regulated by *ATM*, *Chk2*, and *p53* and aims at repairing DNA lesions, which may present as obstacles for DNA replication machinery. Replication is a tightly regulated process and, if compromised, it results in replication stress, which is a central cause of genome instability [50,51]. The intra-S phase checkpoint is regulated by *ATR*, *Chk1*, *DNA-PK*, and *Wee1*. When triggered, this checkpoint delays the replication process to allow the cells to repair and to cope with DNA damage during S phase. The G2/M checkpoint is regulated by *Chk1*, myelin transcription factor 1 (*Myt1*), and *Wee1*, safeguarding genomic stability by preventing cells to exit G2/M cell cycle phase and divide prior repairing existent DNA lesions. The failure to repair DNA damage at G2/M phase followed by an attempt to divide may result in mitotic catastrophe and cell death [51,52].

SSBs are the most common DNA lesions and they are repaired through a process called base excision repair (BER). During BER, SSBs are recognized by poly(ADP-ribose) polymerase (*PARP*), which induces extensive poly-ADP-ribosylation (PARylation) and thereby initiates their repair [53]. In particular, *PARP* recruits the scaffold protein X-ray cross-complementing protein 1 (*XRCC1*) followed by additional enzymes such as DNA polymerase β (*POLβ)* and DNA ligase 3 (*Ligase III*) [54,55]. *POLβ* is responsible for filling in the single-nucleotide gap and preparing the strand for ligation by *Ligase III* [56]. SSBs can indirectly lead to DSB formation if two of them arise in close proximity of each other. Moreover, if left unrepaired, SSBs can be converted into DSBs, ultimately resulting in replication fork stalling and collapse [57]. Other major pathways involved in the maintenance of genomic stability include nucleotide excision repair (NER) and mismatch repair (MMR). The NER pathway removes helix-distorting lesions and primarily copes with UV-induced damage [58]. The MMR pathway corrects mismatched nucleotides, insertions, and deletions acquired during DNA replication [59].

In cancer, the critical balance between the genomic maintenance in malignant cells and the number and partial overlap of DDR pathways (Figure 4) provide an exciting therapeutic opportunity. Because different DNA repair pathways may functionally overlap, and as one pathway can sometimes “back-up” for defects in another, inhibition of pathways presented in a cancer cell should in some cases have a greater impact on the cancer than on normal tissues. This deficiency in the cancer cells can be exploited using an approach termed synthetic lethality. A paradigm for this is provided by drugs targeting the enzyme *PARP1*, which binds SSBs and BER intermediates to facilitate DNA repair processes. Notably, PARP inhibitors are relatively nontoxic to normal cells but are strikingly cytotoxic towards HR-defective cells, particularly those with impaired *BRCA1* or *BRCA2* function [60,61]. In *BRCA*-deficient cells, PARP inhibition drives the collapse of replication forks and the formation of replication-associated DSBs that can only be repaired by HR [45,60,61,62,63].

## 4. DNA Repair Gene Expression in DMGs

The rampant inter- and intra-patient heterogeneity with treatment-resistant cancer stem-like cells (CSCs) at the apex is believed to drive the resistance to existent chemo- and radiotherapy [64,65,66,67,68]. A study performed by Alhajala et al. showed that genes involved in stem cell differentiation, angiogenesis, cell proliferation, and cell growth are upregulated in response to irradiation in pGBM with wild-type H3 [69]. Harutyunyan et al. have reported that the loss of H3K27me2 and H3K27me3 dysregulates the expression of genes involved in stemness and neurogenesis, thereby affecting cell differentiation [37]. Larson et al. have shown that H3.3 K27M mutation alone enhances neural stem cell self-renewal in a genetically engineered mouse model [70]. CSCs were found to constitituvely activate DDR signaling, which is believed to protect them from the insult of DNA damaging agents [67]. In H3K27M-mutated tumors, the dysregulation of stem cell maintanence and aberrant DDR activation represent a vulnerability that may be exploited for their sensitization to DNA damaging therapies such as chemo-irradiation.

The identification of histone mutations in a large proportion of HGG from pediatric and adolescent patients has increased the enthusiasm for targeted combinational approaches to tackle these tumors [6,7]. The first major breakthrough came in 2012, when studies using whole-genome and whole-exome sequencing reported that 80% of DIPG and 35% midline glioblastoma (mGBM) possess mutations in histone H3.3/3.1 and that these mutations, together with the abrogation of cell cycle checkpoints, confer radio-resistance and dismal prognosis [6,7,10,71]. Perturbations in DNA damage repair gene expression in H3K27M mutant tumors were first identified in 2010 by Zarghooni et al., who reported a loss of heterozygosity (LOH) of a number of DNA repair genes [7]. In 2017, Mackay et al. performed an integrated genomic analysis, which likewise found that 60.7% of the pHGG harbored genetic alterations in the DNA repair pathway, highlighting DNA repair as a potential therapeutic target [11]. Moreover, Mackay et al. found a diverse set of heterozygous mutations in a number of genes involved in error-free HR repair and numerous Fanconi anemia genes [11]. Their analysis identified, among others, amplifications of cyclin D2 (*CCND2*) and *TOP3A* in H3.3K27M DIPG [11]. *CCND2* promotes cell cycle progression by phosphorylation of retinoblastoma (Rb), whereas *TOP3A* is involved in HR [72,73]. It remains unknown as to whether these alterations are involved in the radio-resistance seen in DMGs. A study by Zhang et al. has shown that the loss of H3K27me2 and H3K27me3 reduces the number of *53BP1* foci as well as decreasing the efficiency of NHEJ in human dermal fibroblasts [74]. *ATM* is necessary for the initiation of DDR and DSB repair and was found mutated in 10.7% of H3.3K27M brainstem DMGs [11], but the consequences of such combination on genomic stability and therapeutic resistance remain elusive [75]. *TP53* regulates cell cycle arrest, senescence, apoptosis, autophagy, metabolism, and aging. About 42–77% of DMGs carry the *TP53* mutation, a phenotype that is associated with increased radio-resistance, early relapse, and worsened prognosis compared to *TP53* wild-type DMGs [76,77]. Moreover, mutations in *PPM1D* have been identified in up to 25% of DIPGs [31,78]. *PPM1D* encodes the wild-type p53-induced phosphatase 1 (*Wip1*), which has been shown to be a negative regulator of the DDR by dephosphorylating *ATM* [79], *TP53* [80], *Chk2* [81], *Chk1* [80], and *γH2AX* [82], which together lead to a reduced activation of *TP53*. This could potentially contribute to the radio-resistance in the DMGs harboring the *PPM1D* mutation, as seen for the DMGs harboring the *TP53* mutation [77].

## 5. DNA Repair as a Therapeutic Target to Radio-Sensitize DMGs

**Figure 5:**
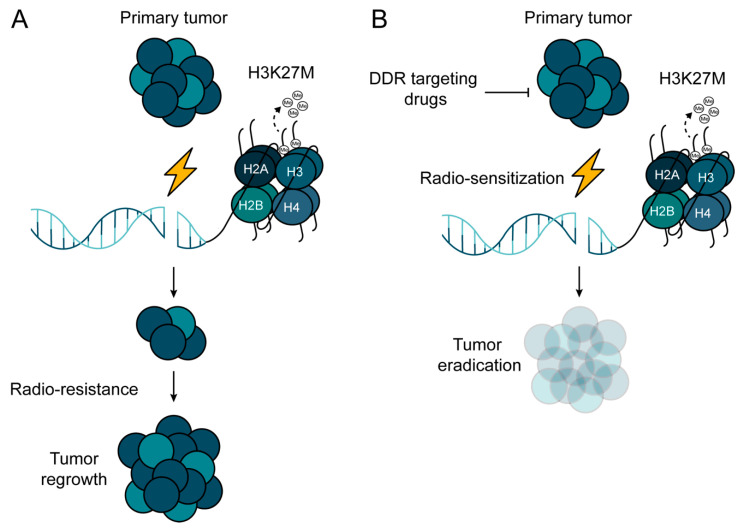
Potential treatment strategy to overcome radio-resistance in H3K27M-mutated pHGG. (**A**) H3K27M mutation confers radio-resistance in pHGG due to the de-regulation of DNA repair machineries. (**B**) Combinational approach, where radiotherapy (RT) is combined with DNA repair inhibitors, may pave the way for overcoming the radio-resistance of H3K27M-mutated pHGG.

Whereas RT has proven to be an effective palliative and curative tool in cancer management, it is not without risk of toxicity to normal tissues [83]. A wide variety of tumor types demonstrate resistance to RT, which limits its utility as a stand-alone treatment modality and stresses the unmet need for the identification and successful validation of radio-sensitizing agents. There have been attempts to improve the outcome of RT with the integration of systemic agents before, during, or after IR in a number of solid malignancies. In DMGs, the number of studies interrogating radio-sensitizing agents is limited. Those studies that have been reported thus far did not conclude a clear clinical benefit compared to the standard of care. Taking the vast inter- and intra-tumoral heterogeneity into consideration, better stratification and identification of patients who might benefit from being given a drug–IR combination is necessary, which can be warranted only through international multi-center collaborative efforts. The fact that H3K27M mutation confers radio-resistance warrants further investigation into using radio- and chemo-sensitizing agents in the therapeutic intervention of H3K27mM-mutated MGs. Using synthetic lethal approaches to H3K27M mutation may provide a rational way to develop novel and highly selective radio-sensitizing strategies (Figure 5).

The overall limited impact of RT in the management of cancer fueled intensive research efforts, leading to pre-clinical and clinical testing of various agents and/or tool compounds. DNA damage-inducing agents such as TMZ, cyclophosphamide, etoposide, and lomustine have been interrogated in regards to their ability to improve the efficacy of RT, with no success. O6-Methyl-guanine-DNA methyltransferase (*MGMT*) promoter methylation is associated with TMZ resistance. In a study of 46 DMGs with confirmed *H3F3A* mutation, none had *MGMT* promoter methylation. Through a series of in vitro experiments, Abe et al. confirmed that epigenetically driven high expression of MGMT is the main reason for TMZ resistance [84,85].

We and others have shown that the DDR is constitutively active in adult malignant gliomas (MG) due to ongoing oxidative and replication stress [86,87] and that the targeting of DDR components may be of high clinical relevance, as evidenced by extended survival of tumor-bearing animals in in vivo studies [88,89]. A number of reports highlighted the aberrant expression of DNA repair factors in DMGs, indicating impaired DNA repair [11,90]; however, the extent of DDR activation and the functionality of DNA repair mechanisms in DMGs remains elusive. A subset of DMGs has been found to overexpress *PARP1*, and its inhibition has been studied as a potential therapeutic intervention in DMGs, resulting in radio-sensitization and anti-tumor efficacy both in vitro and in vivo. Unfortunately, the clinical relevance of these findings is tainted by the model choice for some of the key experiments shown in this particular study. The authors used pediatric high-grade astrocytoma cells instead of DMG cells and injected these into the cortex instead of the pons in their in vivo proof-of-concept study [38,91].

A study by Akamandisa et al. (2019) showed that targeting *PPM1D* effectively eradicates *PPM1D* mutant DMGs in vitro and in vivo, especially when combined with irradiation [92]. In the presence of DNA damage, DDR activates cell cycle checkpoints to prevent cells from entering mitosis. *PLK1* is one of the DNA damage checkpoints [93], while also controlling DNA repair [94]. A study by Amani et al. concluded that targeting *PLK1* with small molecule inhibitors, in combination with IR, warrants further investigation on the basis of their in vitro evidence of reduced proliferative index and increased apoptosis in DMGs treated with *PLK1* inhibitor BI 6727 in combination with IR [95]. In DMGs, *p53* function is often compromised by loss-of-function mutations, genomic deletions, or indirectly as a result of *PPM1D* mutations [10,38]. Nikolaev et al. (2020) showed that a combination of eprenetapopt (also known as APR-246 or PRIMA-1MET, a small organic molecular that induces oxidative stress and reactivates mutant *p53*) and histone lysine demethylase inhibitor VIII (GSK-J4, a cell permeable prodrug of GSK-J1, which is a selective inhibitor of the H3K27 histone demethylase of Jumonji histone demethylases) markedly sensitizes DMGs to IR [96]. The mammalian target of rapamycin (mTOR) signaling pathway is one of the key oncogenic drivers in DMGs. A growing body of evidence suggests a close link with DDR and many factors important for the DDR were found to be involved in the mTOR pathway [97]. One study found that a dual mTOR kinase inhibitor sapanisertib (TAK228) inhibited tumorigenicity and enhanced the effect of RT in DMG, supporting this connection [98]. Despite the evidence from pre-clinical studies supporting radio-sensitizing strategies, the number of currently active clinical trials exploring the impact of novel agents in combination with IR is limited. Among the reasons that preclude successful translation is the lack of in vivo validation using patient-derived xenograft models, which are more representative of original tumor architecture than commercially available stable cell lines [99].

An example of a putative radio-sensitizer assessed both in a pre-clinical study and a clinical trial is the selective *Wee1* inhibitor adavosertib (MK-1775). *Wee1* is a critical component of the G2/M cell cycle checkpoint machinery, which prevents mitotic entry in the presence of DNA damage [100]. In 2013, Mueller et al. reported *Wee1* to be overexpressed in DIPGs and proposed the use of the *Wee1* inhibitor in combination with IR for the treatment of DIPG [41]. The NCT01922076 study, which is currently running, is a phase I trial assessing the safety, toxicity, and maximum tolerated dose (MTD) for adavosertib administered concurrently with radiation therapy in children with newly diagnosed DIPGs.

B lymphoma Moloney murine leukemia virus insertion region 1 (BMI-1), a core member of polycomb repressive complex 1 (PRC1), has been intensely investigated in the field of cancer epigenetics and DNA repair for decades. The recruitment of *BMI-1* to sites of DNA DSBs is critical for efficient HR repair [101]. Kumar et al. have shown that *BMI-1* is highly expressed in DIPGs compared to normal tissue and that downregulation in vitro leads to inhibition of neurosphere formation, migration, self-renewal, and cell-cycle signaling. Importantly, this study reported that the targeted inhibition of *BMI-1* sensitizes DIPG cells to radiomimetic drug-induced DNA damage [102]. The safety of PTC596 (an orally active inhibitor of *BMI-1*) taken during radiation in children with newly diagnosed DMGs and HGG is currently being investigated in a phase Ib clinical trial (NCT03605550).

A study by Werbrouck et al. has shown promising data on the efficacy of the *Chk1* inhibitor LY2606368 in combination with IR in TP53-mutated DMGs [77]. LY2606368 is also being investigated within a phase I clinical in refractory pediatric tumors including DMGs as a monotherapy, with the aim being to analyze putative biomarkers and estimate the MTD as a primary objective (NCT02808650) [77].

A recently initiated clinical trial in newly diagnosed DIPGs was designed to explore the impact of the chemotherapeutic agent gemcitabine as a monotherapy (NCT02992015) [103]. Gemcitabine (difluorodeoxycytidine; dFdCyd) inhibits DNA replication and was found to radio-sensitize a wide range of cancer cell models in vitro [104,105]. Although the mechanism of sensitization is not yet clear, recent evidence suggests that gemcitabine lowers the threshold for radiation-induced apoptosis [106]. An open-label, single-arm phase I/II trial in newly diagnosed DMGs explored the potential and safety of gemcitabine administered concomitantly with RT. This study demonstrated that weekly gemcitabine concomitant with RT is safe. Unfortunately, due to a limited number of patients included, this study failed to draw any conclusions in terms of the efficacy of this combination [107].

Improved mechanistic understanding on the role of EGFR signaling in the onset of DMGs, prompted the use of nimotuzumab, humanized the immunoglobulin G1 (IgG1) anti-EGFR monoclonal antibody in DMGs. Assuming a potential synergy with both RT and vinorelbine (a semi-synthetic vinca alkaloid), Massimino et al. (2014) launched a non-randomized, open label phase II pilot study with the aim of assessing the safety of this combination. The combination was well tolerated [108]. In 2019, a multinational phase III trial evaluated the safety and efficacy of nimotuzumab in combination with RT in newly diagnosed DMGs [109]. This study concluded that concomitant treatment with RT and nimotuzumab was feasible in an outpatient setting, but the progression-free survival and overall survival were not superior compared to the results achieved with RT and intensive chemotherapy in a hospitalized setting [109].

## 6. Conclusions

With little improvement in the outcome of H3K27M-mutated glioma (DMGs) over the past four decades, median survival rates remain at approximately 9 to 11 months. There is an unmet need for novel treatment strategies in this patient group. We believe that the H3K27M mutation-driven dysregulation of DDR pathways intrinsic to DMGs represents a unique vulnerability that can be leveraged for their sensitization to DNA damaging therapies such as IR. Unfortunately, our understanding of the functional interplay between DNA repair and radio-resistance in DMGs remains poor. A better comprehension of molecular mechanisms underpinning DNA repair, radio-resistance, and the efficacy, as well as safety of radio-sensitizing strategies, represents a critical step preceding a successful therapeutic intervention and improved survival of DMG patients.

## Figures and Tables

**Table 1:** Concurrent mutations in the *H3 K27M* subtype [8,9].

Alteration	Gene
Mutations	Tumor suppressor protein p53 (*TP53*)
Mutations	ATRX chromatin remodeler (*ATRX*)
Mutations	Neurofibromin 1 (*NF1*)
Mutations	Protein phosphatase magnesium-dependent 1 delta (*PPM1D*)
Mutations	Phosphatidylinositol-4,5-bisphosphate 3-kinase catalytic subunit alpha (*PIK3CA*)
Promoter mutations	Telomerase reverse transcriptase (*TERT*)
Gene fusions	Neurotrophic receptor tyrosine kinase (*NTRK*)
Upregulation	Paired box 3 (*PAX3*)
Upregulation	Interleukin-13 receptor subunit alpha-2 (*IL-13RA2*)
Upregulation	Poly(ADP-ribose) polymerase 1 (*PARP1*)
Deletions	Phosphatase and tensin homolog (*PTEN*)
Amplification	Cyclin D1/2/3 (*CCND1/2/3*)
Amplification	Cyclin-dependent kinase 4/6 (*CDK4/6*)
Amplification	MET proto-oncogene receptor tyrosine kinase (*MET*)
